# Human beta defensin-2 protects the epithelial barrier during methicillin-resistant *Staphylococcus aureus* infection in chronic rhinosinusitis with nasal polyps

**DOI:** 10.3389/fcimb.2025.1551080

**Published:** 2025-05-09

**Authors:** Tengfei Tian, Szuyao Hsu, Qin Sun, Yang Shi, Xianyang Hu, Yang Wu, Keqing Zhao, Chunquan Zheng

**Affiliations:** ^1^ Department of Otolaryngology, Eye & ENT Hospital, Fudan University, Shanghai, China; ^2^ Laboratory for Reproductive Immunology, Shanghai Key Laboratory of Female Reproductive Endocrine Related Diseases, Hospital of Obstetrics and Gynecology, Shanghai Medical College of Fudan University, Shanghai, China; ^3^ Key Laboratory of Medical Molecular Virology (MOE/NHC/CAMS), Department of Medical Microbiology and Parasitology, School of Basic Medical Sciences, Shanghai Medical College of Fudan University, Shanghai, China

**Keywords:** human beta defensin-2, methicillin-resistant *Staphylococcus aureus*, chronic rhinosinusitis with nasal polyps, epithelial barrier, tight junctions

## Abstract

**Objective:**

We investigated the effect of human beta defensin-2 (hBD-2) on nasal epithelial barrier function with methicillin-resistant *Staphylococcus aureus* (MRSA) infection in chronic rhinosinusitis with nasal polyps (CRSwNP).

**Methods:**

The expression of hBD-2 was measured in nasal polyps (NPs) from CRSwNP. MRSA was treated with different concentrations of hBD-2 to assess the invasive ability. Primary human nasal epithelial cells (HNECs) cultured at the air–liquid interface (ALI) were pre-incubated with or without hBD-2 prior to MRSA infection. The cell viability, the epithelial cell integrity, and the tight junction (TJ) expression were evaluated.

**Results:**

The expression of hBD-2 in the CRSwNP group was higher than that in the control group. In addition, the hBD-2 protein was negatively correlated with the Lund–Mackay CT score and was positively correlated with the neutrophil levels in CRSwNP. The presence of hBD-2 significantly reduced the invasive ability of MRSA in HNECs. MRSA decreased the epithelial cell integrity by diminishing the protein expression of occludin and zonula occludens-1 (ZO-1). Furthermore, hBD-2 prevented the MRSA-induced barrier disruption by increasing the mucosal permeability and the expression of occludin and ZO-1.

**Conclusion:**

The results suggest that hBD-2 may partially attenuate the epithelial barrier disruption induced by MRSA, indicating the protective effect of hBD-2 on *S. aureus* infection.

## 1 Introduction

Chronic rhinosinusitis (CRS) is a common sinonasal mucosal inflammatory disease characterized by significant nasal obstruction, craniofacial pain, decreased sense of smell, and inflammation of the mucosal lining of the sinuses, affecting 7%–27% of the adult population every year (Bachert et al., 2020; Sedaghat et al., 2022; Shi et al., 2015). It comprises a group of heterogeneous diseases that can be classified into binary phenotypes based on the presence (chronic rhinosinusitis with nasal polyps, CRSwNP) or absence (chronic rhinosinusitis without nasal polyps, CRSsNP) of nasal polyps (NPs) (Fokkens et al., 2020). *Staphylococcus aureus S. aureus*is one of the most common pathogens in CRS (Liang et al., 2023). Nasal carriage of *S. aureus* occurs in up to 70% of patients with CRSwNP. *S. aureus* colonization of the upper airways appears to be a significant external trigger in the pathogenesis of CRS. It may exhibit a series of mechanisms to invade the immune system, including the secretion of enterotoxins that impact the ciliary structure and function, the formation of biofilms to isolate the pathogens from immunocytes, and the production of virulence factors that target the epithelial barrier (Foreman et al., 2012; Jervis-Bardy et al., 2009; Thammavongsa et al., 2015). Methicillin-resistant *S. aureus* (MRSA) is associated with recalcitrant disease, antibiotic tolerance, and poor postoperative outcomes (Al-Asousi et al., 2017). However, the mechanism of action of MRSA in the disease remains to be elucidated.

The nasal mucosal epithelium collaborates with neutrophils against microbial invasion, acting as the physical barrier of the first-line defense. Numerous studies have shown the importance of epithelial barrier function in CRS and other airway diseases (Barham et al., 2015; Hellings and Steelant, 2020). Dysregulation of the sinus epithelium could lead to a series of morphological changes, including mucociliary dysfunction, mucus overproduction, and disrupted maintenance of the air–liquid surface and ion transport (Schleimer et al., 2007). Furthermore, the epithelial integrity, which is maintained by intercellular protein complexes called “tight junctions” (TJs), is undermined (Pothoven et al., 2015). TJs are composed of transmembrane proteins including claudin, occludin, junctional adhesion molecules, and intracellular scaffolding proteins anchored to the actin cytoskeleton, such as zonula occludens (Balda and Matter, 2016; Sturgeon and Fasano, 2016). These protein complexes regulate the epithelial impermeability against pathogens and foreign particles, ions, and certain molecules through the paracellular space (Jang et al., 2002). In the context of the nasal epithelium, TJ proteins are downregulated in mature polyps, resulting in barrier dysfunction and the entry of pathogens (Nur et al., 2021; Rogers et al., 2011).

In addition to the role of the physical barrier against microbes, antimicrobial peptides (AMPs) produced at the epithelial surfaces have recently been identified as important factors that enhance the barrier function (Chung and Khanum, 2017). Human defensins comprise one of the major AMPs, classified into alpha-defensins (human neutrophil peptides, hNPs) and beta-defensins (hBDs) (Luo and Song, 2021). They are a group of cationic polypeptide molecules that were originally reported to exert antimicrobial activity and regulation of the microbiota composition (Supp et al., 2004). These hBDs can be expressed either constitutively or induced in various tissues and cell types in response to microbial challenge and the production of inflammatory mediators (Schneider et al., 2005). Recently, it has become evident that hBDs are associated with the initiation and development of various inflammatory disorders and diseases (Fusco et al., 2021; Kiatsurayanon et al., 2014). Among them, human β-defensin 2 (hBD-2) have recently been reported to enhance the skin integrity by increasing the expression of TJ proteins and the transepithelial electrical resistance (Wang et al., 2017). Moreover, the hBD-2 secreted by intestinal epithelial cells was found to reduce the invasive ability of *Candida albicans*, in addition to upregulating the expression of TJs upon infection (Fusco et al., 2021).

Despite the great involvement of the microbiota, particularly *S. aureus*, in CRS and its potential effect on barrier dysfunction, the role and the mechanism of hBD-2 in regulating the epithelial barrier function (Sedaghat et al., 2022) in CRS have not yet been investigated. We hypothesize that hBD-2 might protect the human nasal epithelial layer upon *S. aureus* invasion by upregulating the expression of TJs and by downregulating the invasive ability of *S. aureus* in CRS. The results should enable us to better understand the mechanisms by which hBD-2 exerts its antimicrobial function in the sinonasal environment during infection.

## 2 Materials and methods

### 2.1 Subjects

NP tissues were obtained from patients with CRSwNP who underwent functional endoscopic sinus surgery. We excluded patients who: a) were older than 70 or younger than 18 years; b) had cystic fibrosis, immunodeficiency, aspirin intolerance, Churg–Strauss syndrome, asthma, fungal sinusitis, and other nasal diseases; and c) had infectious or tumors, serious systemic diseases, and mental diseases. The sinus opacity was scored radiologically, and the severity and the size of NPs were assessed with the Lund–Mackay CT score. The Lund–Mackay CT score was calculated by grading each sinus (i.e., anterior ethmoid, posterior ethmoid, maxillary, sphenoid, frontal, and the ostiomeatal complex) on a scale of 0–2 (0, no opacification; 1, partial opacification; and 2, total opacification), with total scores ranging from 0 to 24 per patient (Lund and Kennedy, 1997). The normal control inferior turbinate (IT) tissues were obtained from patients who underwent septoplasty due to anatomic variations and who did not have sinonasal inflammatory disease. The clinicodemographic characteristics of all subjects are presented in **Table 1**. This study was approved by the Ethics Committee of the Eye & ENT Hospital of Fudan University (2024-YS-137), and all subjects provided informed consent prior to sample collection.

**Table 1:** Clinical characteristics of the subjects.

Characteristic	Control (*n* = 25)	CRSwNP (*n* = 35)	*P*-value
Age (years)	40.3 ± 7.6	46.8 ± 11.0	–
Sex (M/F)	18/7	22/13	–
Allergic rhinitis, *n* (%)	1 (7.7)	6 (17.1)	–
Asthma, *n* (%)	0 (0)	1 (3.7)	–
Lund–Mackay CT score, median (mean ± SD)	–	11.3 ± 5.0	–
Blood NEU count (×10^9^ cells/ml)	4.3 ± 1.1	5.5 ± 1.8	<0.001
NEU percentage (%)	55.2 ± 7.9	62.7 ± 8.3	<0.001

*CRSwNP*, chronic rhinosinusitis with nasal polyps; *M*, male; *F*, female; *NEU*, neutrophil.

### 2.2 Immunofluorescence staining

Paraffin sections of the nasal biopsy specimens and cultured human nasal epithelial cells (HNECs) were fixed with 4% paraformaldehyde and then blocked with 10% normal goat serum for 30 min at room temperature. They were then incubated with anti-occludin (1:200; Abcam, Cambridge, UK), anti-ZO-1 (1:100; Abcam, Cambridge, UK), and anti-hBD-2 (1:400; Santa Cruz, Dallas, TX, USA) primary antibody solutions overnight at 4°C, followed by 1–2 h incubation with Alexa Fluor 594-conjugated secondary antibodies in the dark at 37°C. The coverslips were mounted on slides using a SlowFade™ Gold antifade reagent with DAPI. The slides were analyzed using confocal microscopy (Leica Microsystems, Wetzlar, Germany). Images were processed with ImageJ software (National Institutes of Health, Bethesda, MD, USA).

### 2.3 Total fluorescence intensity evaluation

The protein expression levels were analyzed using ImageJ software (National Institutes of Health, Bethesda, MD, USA) by calculating the value of the positively stained area and the mean fluorescence intensity for each marker. The total fluorescence intensity (TFI) measurements were performed by multiplying the positive area by the mean fluorescence intensity and then corrected by subtracting the background autofluorescence.

### 2.4 RNA isolation and RT-qPCR analysis

The RNA levels of hBD-2 and the virulence-related genes of *S. aureus* (*hla* and *coa*) were determined with reverse transcription quantitative PCR (RT-qPCR). Frozen nasal tissues and cultured HNECs were disrupted in TRIZol reagent (Takara, Kusatsu, Japan), and total RNA was purified according to the manufacturer’s instructions. Of the total RNA, 1 μg was reverse-transcribed into cDNA using the Maxima Reverse Transcriptase Kit (AG, Hunan, China) according to the manufacturer’s protocol. Overnight cultures of *S. aureus* USA500 strains were diluted 1:200 with tryptic soy broth (TSB) containing hBD-2 (5 μg/ml) and then inoculated at 37°C. The TSB without hBD-2 was used as a control. After 16 h, total RNA was extracted for RT-qPCR. The total RNA was then purified using the RNeasy Plus Mini Kit (QIAGEN, Berlin, Germany) following the manufacturer’s instructions. RT-qPCR was performed using the Mastercycler RealPlex System (Eppendorf AG, Hamburg, Germany) with SYBR Premix Taq (AG, Hunan, China). The relative gene expression was calculated using the comparative 2^−ΔΔCt^ method. *GAPDH* (glyceraldehyde 3-phosphate dehydrogenase) and *gyrB* (DNA gyrase subunit B gene) were used as the housekeeping genes for gene normalization. The primers used for the RT-qPCR in this study are listed in **Supplementary Table S1**. RT-qPCR was performed in triplicate at least three times.

### 2.5 Isolation and culture of primary human nasal epithelial cells

Primary human nasal epithelial cells (pHNECs) were isolated from the above-mentioned nasal tissues. Briefly, nasal samples were washed three times using Dulbecco’s phosphate-buffered saline (DPBS) (Ca^2+^/Mg^2+^-free) supplemented with 1% penicillin, streptomycin, and amphotericin B. Subsequently, the nasal tissues were digested with 0.1% Protease XIV (Sigma, St. Louis, MO, USA) overnight at 4°C. After cell filtration using 70-μm strainers, all cells were washed with Dulbecco’s modified Eagle’s medium (DMEM)/F12 (Gibco, Waltham, MA, USA) and centrifuged at 300 RCF (relative centrifugal force) for 5 min. Thereafter, the cells were cultured in PneumaCult-Ex Medium (STEMCELL, Vancouver, Canada) at 37°C with 5% CO_2_ atmosphere. After reaching approximately 80% confluence, the cells were digested with Accutase (STEMCELL, Vancouver, Canada) for approximately 5 min and then seeded on a Transwell chamber (0.4-μm pore size; Corning, Corning, NY, USA). To induce cell differentiation at the air–liquid interface (ALI) culture, the medium at the basal side of Transwell was replaced by a PneumaCult-ALI Medium kit (STEMCELL, Vancouver, Canada) and the medium at the apical side removed. HNECs were fully differentiated after 24-day culture in ALI. At the end of this time, the HNECs were pre-incubated with or without hBD-2 protein for 2 h. Subsequently, the HNECs were infected with USA500 at an exponential growth phase at a multiplicity of infection (MOI) of 1:10 cells for 4 h. After infection, the cells were harvested for further analysis.

### 2.6 Growth curves of *S. aureus*

Overnight cultures of *S. aureus* USA500 were centrifuged. The liquid cultures of USA500 were sub-cultured (1:200) in fresh TSB medium and hDB-2 (Santa Cruz, Dallas, TX, USA). The bacterial solution without hDB-2 was used as a positive control, and the effect of the derivatives on bacterial growth was automatically monitored every hour by measuring the optical density at 600 nm (OD_600_) for 24 h. The absorbance *versus* time of each strain was plotted.

### 2.7 *S. aureus* adhesion and internalization assay

For the adhesion assay, the pHNECs were lysed by adding 1 ml of cold 0.1% Triton-X100 and were plated in serial dilutions on tryptic soy agar (TSA) for 24 h at 37°C to identify the viable intracellular bacteria by counting the colony forming units (CFU) per milliliter. For the invasion assay, after the first 4 h of incubation, the pHNEC supernatants containing bacteria were removed and replaced with fresh medium containing 40 μg/ml lysostaphin and 200 μg/ml gentamicin for an additional 30 min at 37°C. Subsequently, the cells were lysed and the CFU per milliliter determined.

### 2.8 Epithelial cell invasion assay

HNECs were cultured for 24 days in 24-well Transwell chambers (8-mm pore size; Corning, Corning, NY, USA). Thereafter, the cells were infected with bacteria at a MOI of 10:1 and were incubated at 37°C with 5% CO_2_ for 4 h. Subsequently, with the aim to identify the viable bacteria able to cross the barrier, the medium present in the basolateral side was plated in serial dilutions on TSA (SAB-Oxoid, Lenexa, KS, USA) for 24 h at 37°C by counting the CFU per milliliter.

### 2.9 Measurement of transepithelial electrical resistance and epithelial permeability

The barrier function of the HNEC cultures was measured using two methods: transepithelial electrical resistance (TEER) and fluorescein isothiocyanate (FITC)–dextran flux. Fully differentiated HNECs were co-incubated with or without recombinant hBD-2 (Santa Cruz, Dallas, TX, USA) for 48 h and then hBD-2 was removed. The USA500 treatment and TEER assays were performed immediately every hour to measures the resistance of the cell layer. TEER was determined with a Millicell-ERS system (Millipore, Billerica, MA, USA). The FITC–dextran measurements commenced immediately after 4 h of exposure to USA500. Dextran flux was measured by placing 10 kDa FITC-conjugated dextran (Sigma, St. Louis, MO, USA) in the upper chamber of the Transwell chamber for 30 min to assess barrier function, after which the supernatants were harvested from the lower chamber. A Molecular Devices Spectramax Gemini EM fluorimeter (Molecular Devices, San Jose, CA, USA) was used to measure the amount of FITC–dextran that crossed through the cell layer with excitation at 495 nm and emission at 520 nm.

### 2.10 CCK-8 assay and LDH release assay

Cell viability was measured using the CCK-8 Kit (Beyotime, Shanghai, China) according to the manufacturer’s instructions. Briefly, 10 μl of the CCK-8 solution was added to each well for 1 h incubation at 37°C, and the absorbance at 450 nm was measured using a microplate reader. Cytotoxicity was measured by the release of lactate dehydrogenase (LDH) in the supernatant according to the manufacturer’s instructions. Briefly, the cells were seeded in a 96-well plate. After incubation at 37°C, the cells were centrifuged at 4°C for 5 min and the supernatant transferred into a new 96-well plate. The supernatant was mixed with a 100-μl LDH reaction for 1 h incubation. The results were obtained using a microplate reader with excitation at 450 nm and emission at 650 nm.

### 2.11 Statistical analysis

Statistical analyses were performed in GraphPad Prism software 9.0 (GraphPad Software, San Diego, CA, USA). All data were presented as the mean ± standard error of the mean (SEM). Comparisons between two groups were performed with an unpaired Student’s *t*-test or the Mann–Whitney test. Experiments with multiple comparisons were evaluated using one-way ANOVA or the Kruskal–Wallis test, while *post-hoc* pairwise comparisons were performed using Dunn’s test with Bonferroni correction. Correlations were determined using Spearman’s analysis. A *p*-value less than 0.05 was considered significant.

## 3 Results

### 3.1 The hBD-2 levels were increased in patients with CRSwNP

**Figure 1:**
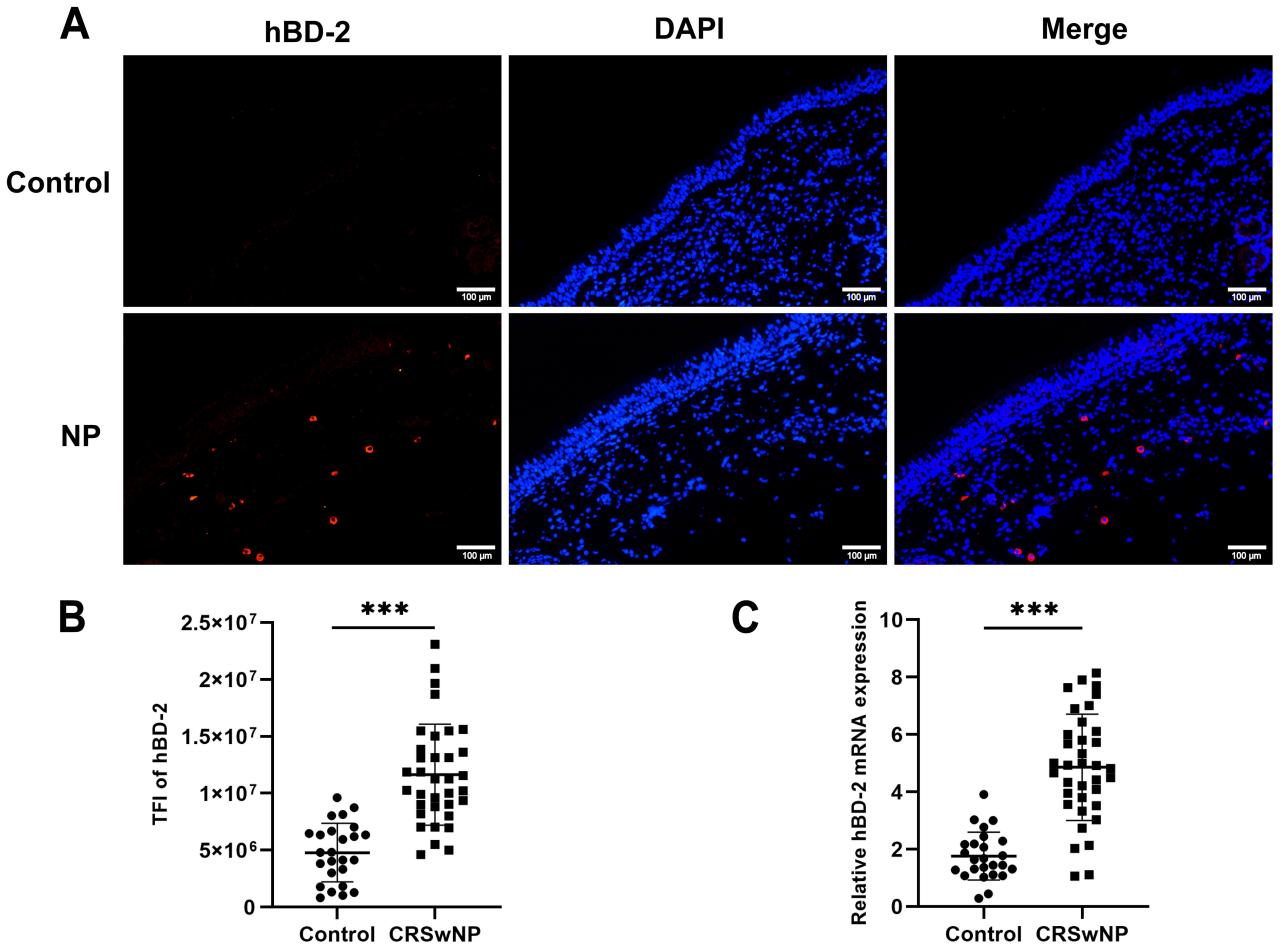
Expression levels of human hBD-2 in the nasal tissues of the control and of CRSwNP. **(A)** Representative immunofluorescence images of the hBD-2 protein (*red color*) in patients with CRSwNP compared with the control. **(B)** Quantitative analysis of the TFI of hBD-2 in patients with CRSwNP compared with the control. **(C)** Expression of hBD-2 mRNA in patients with CRSwNP compared with the control. *TFI*, TFI. Data are expressed as the mean ± SD. ***p < 0.001. *Scale bar*, 20 μm.

To explore whether the expression of hBD-2 is elevated in the nasal epithelium in CRSwNP, immunofluorescence staining was conducted on nasal tissues from healthy controls and from patients with CRSwNP. As shown in **Figure 1A**, the expression of hBD-2 was significantly higher in the nasal epithelium of patients with CRSwNP than in healthy controls. The TFI results of hBD-2 (2.6-fold, *p* < 0.001) (**Figure 1B**) was significantly higher in CRSwNP than in control subjects. In accordance with the staining results, the mRNA expression of hBD-2 in patients with CRSwNP was significantly higher than that in healthy controls (*p* < 0.001) (**Figure 1C**).

### 3.2 Correlation between elevated hBD-2 and CRSwNP

**Figure 2:**
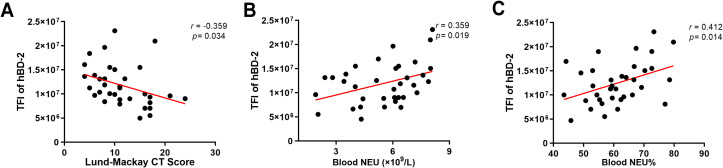
Correlation between human beta defensin-2 (hBD-2) protein expression and chronic rhinosinusitis with nasal polyps (CRSwNP) severity. **(A)** The hBD-2 protein levels were negatively correlated with the Lund–Mackay CT score. **(B, C)** The hBD-2 protein levels were positively correlated with the peripheral blood neutrophil counts (×10^9^/L) and the peripheral blood neutrophil percentage (NEU%). Data were analyzed using nonparametric Spearman’s correlation. *NEU*, neutrophil; *TFI*, total fluorescence intensity.

To determine whether the levels of hBD-2 are related to the clinical disease severity, the Lund–Mackay CT score, which measures the degree of sinus opacification based on computed tomography imaging, was calculated. It was found that the hBD-2 protein expression, referred to as the total immunofluorescence intensity of hBD-2, was negatively correlated with the Lund–Mackay CT score (**Figure 2A**). In addition, the absolute count and the percentage of peripheral blood neutrophils were calculated to explore whether the expression of hBD-2 was correlated with neutrophilic inflammation. The results showed that the protein levels of hBD-2 were positively correlated with the neutrophil count and percentage in CRSwNP (**Figures 2B, C**).

### 3.3 hBD-2 inhibits the growth of *S. aureus*

**Figure 3:**
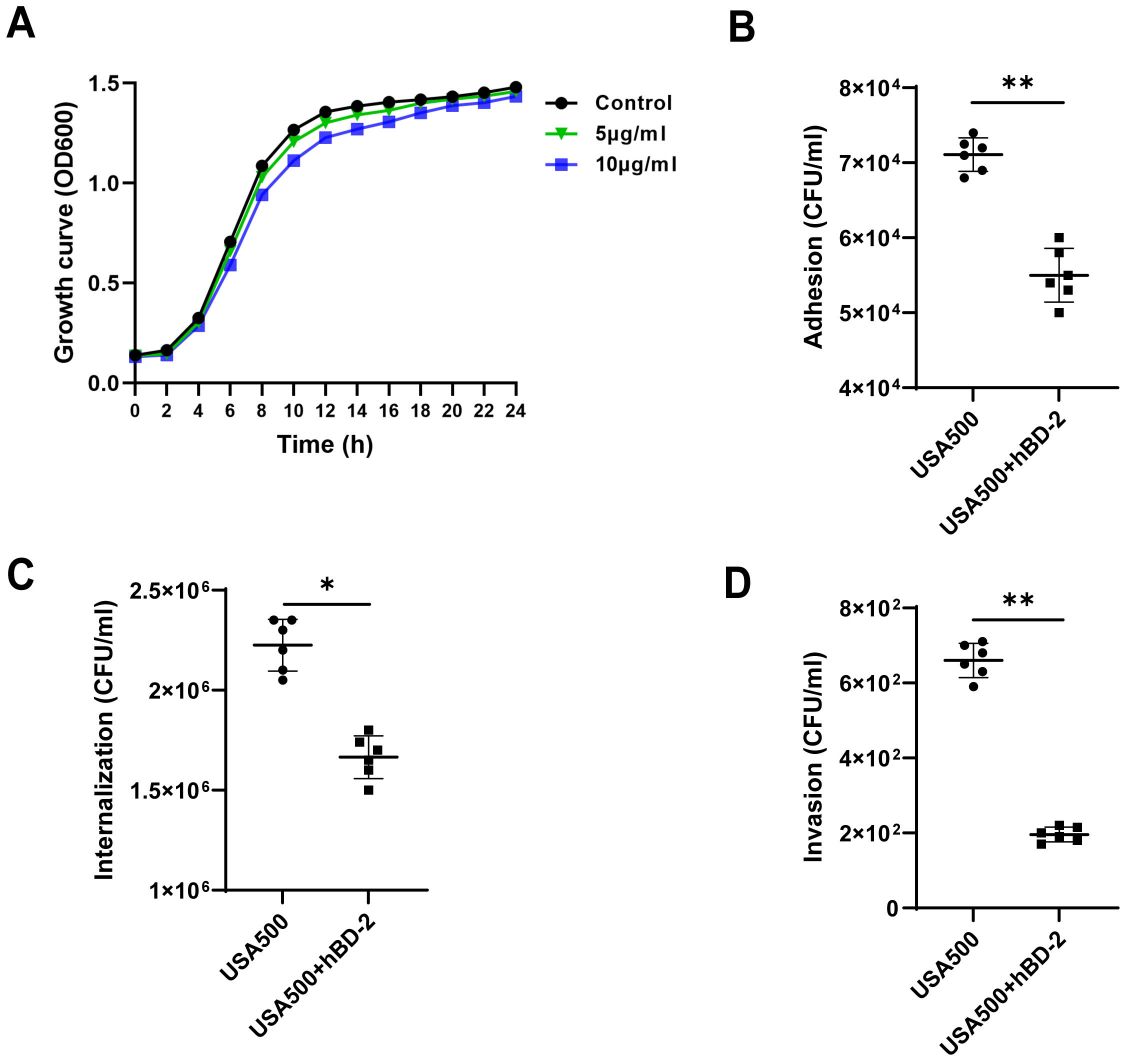
Effect of human beta defensin-2 (hBD-2) on the growth curves and invasion profile of *Staphylococcus aureus*. **(A)** *S. aureus* USA500 was treated with hBD-2 at 5 and 10 μg for 24 h. The growth of their planktonic cells was determined by optical density at 600 nm (OD_600_). **(B, C)** *S. aureus* USA500 adhesion and internalization assay. The number of bacteria was determined by host cell lysis, plating, and counting of the colony forming units (CFU) per milliliter. **(D)** *S. aureus* USA500 Transwell invasion assay. The number of bacteria able to cross the nasal epithelial barrier was determined by plating the yeast in the basolateral site and counting the CFU per milliliter. Data are expressed as the mean ± SD. *p < 0.05, **p < 0.01.

Since hBD-2, as antimicrobial peptides, exhibit a broad-spectrum function against pathogens, we next to explored the effect of hBD-2 on the growth of *S. aureus* USA500 strains. Bacterial growth of USA500 was measured after 24-hour treatment with different concentrations of recombinant hBD-2. We found that hBD-2 at (5 or 10 μg/ml) had little effect on USA500 growth (**Figure 3A**). In particular, USA500 treated with 10 μg/ml of hBD-2 suppressed the growth more compared to 5 μg/ml of hBD-2 treatment. In summary, hBD-2 demonstrates a concentration-dependent ability to suppress the growth of *S. aureus*. Subsequently, the RNA levels of virulence-related genes (*hla* and *coa*) of *S. aureus* were also detected. As shown in **Supplementary Figure S1**, when the *S. aureus* USA500 strains were treated by hBD-2 (5μg/ml) for 16 h, the *hla* and *coa* RNA levels were significantly decreased (*P* < 0.01 and (*P* < 0.05).

### 3.4 Effect of hBD-2 on *S. aureus* adhesion and internalization

We chose 5 μg/ml as a suitable concentration of hBD-2 for the culture of *S. aureus* USA500 strains for the remainder of the experiments. The adhesion and the internalization ability of hBD-2 were assessed using HNECs infected with USA500. The adhesion of USA500 (~7.11 ± 0.22 × 10^4^ CFU/ml) was significantly reduced after pre-incubation with hBD-2 (~5.50 ± 0.35 × 10^4^ CFU/ml), with an initial inoculum of ~4 × 10^8^ CFU/ml (*p* < 0.01) (**Figure 3B**). Moreover, the invasive ability of USA500 was significantly reduced by hBD-2 pre-incubation (~1.66 ± 0.10 × 10^6^ CFU/ml) with respect to untreated USA500 (~2.2 ± 0.13 × 10^6^ CFU/ml) in the same concentration of inoculum (*p* < 0.05) (**Figure 3C**).

The invasive ability of hBD-2 on the human nasal epithelial barrier was then confirmed by USA500 infection to ALI monolayer cultures of HNECs using the Transwell assay. The results showed that USA500 was able to cross the epithelial barrier with an invasion efficiency of 660.0 ± 45.6 CFU/ml without hBD-2 treatment. However, the degree of invasiveness decreased to 195.8 ± 19.6 CFU/ml after hBD-2 treatment (*p* < 0.01) (**Figure 3D**). Overall, hBD-2 enhanced the resistance of nasal epithelial cells against *S. aureus* invasion.

### 3.5 hBD-2 ameliorates *S. aureus*-induced cytotoxicity

**Figure 4:**
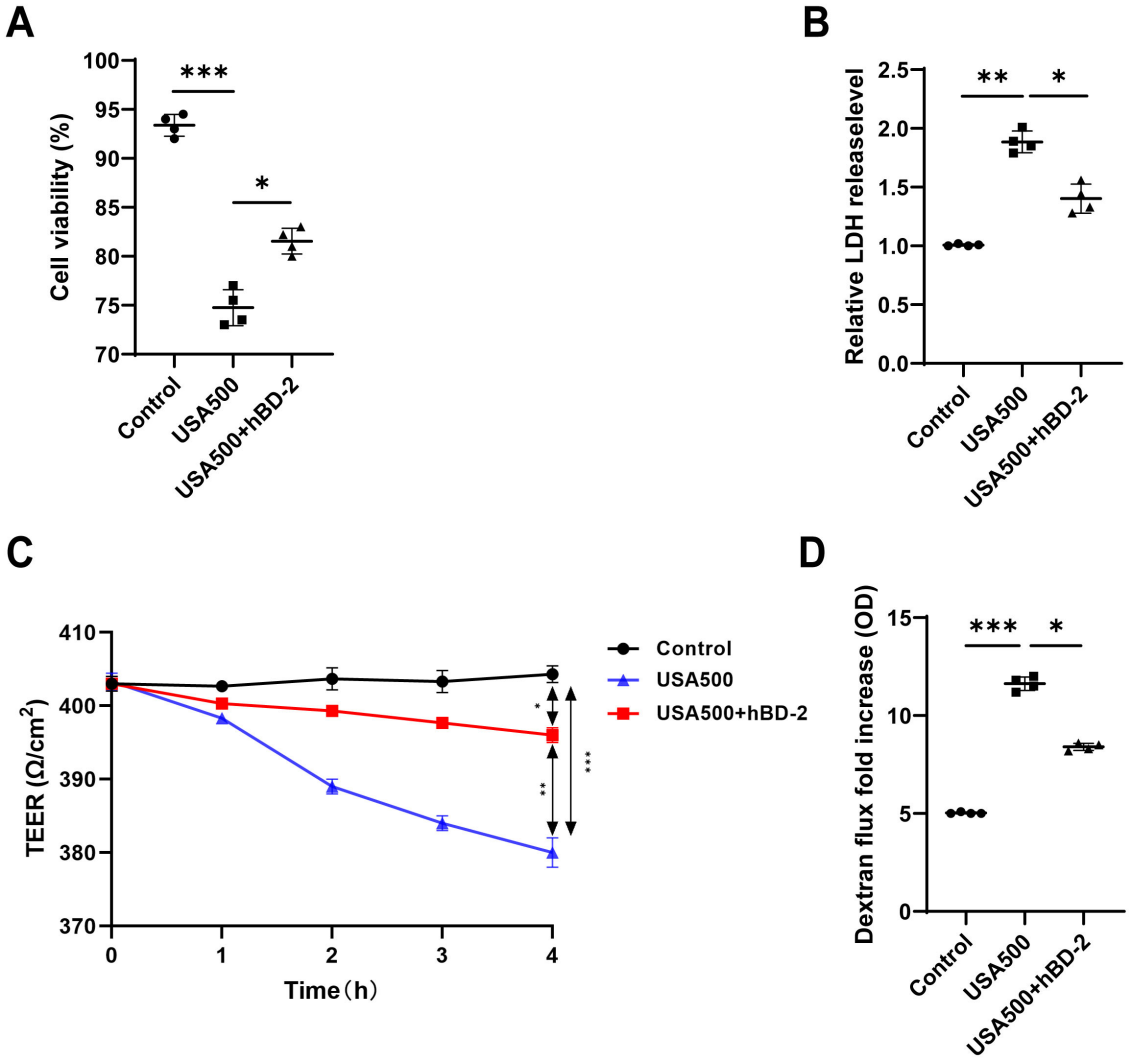
The *Staphylococcus aureus*-induced barrier function was suppressed by human beta defensin-2 (hBD-2) in human nasal epithelial cells (HNECs). **(A, B)** The viability of HNECs was measured using the CCK-8 assay **(A)** and the lactate dehydrogenase (LDH) levels **(B)**. **(C, D)** Epithelial permeability **(C)** and transepithelial electrical resistance **(D)** in HNECs stimulated with *S. aureus* USA500 with and without hBD-2 protein. Data are expressed as the mean ± SD. *p < 0.05, **p < 0.01, ***p < 0.001. *TEER*, transepithelial electrical resistance; *LDH*, lactate dehydrogenase.

To investigate the effect of hBD-2 treatment on cell viability and cytotoxicity in the nasal epithelium layer, HNECs were pre-incubated with and without hBD-2 for 48 h before infection with USA500 *in vitro*. The cell viability of HNECs was significantly decreased upon USA500 infection compared with the uninfected cells, but partially recovered after hBD-2 treatment (**Figure 4A**). Consistently, the LDH levels in the supernatants of USA500-treated cells were considerably increased when compared with the control group, and hBD-2 treatment partially diminished the effect (**Figure 4B**). Our results showed that the decrease in cell viability induced by USA500 was attenuated by treatment with hBD-2. Consistently, treatment with hBD-2 significantly mitigated the *S. aureus*-induced cell death in HNECs, suggesting that the hBD-2 protein may play a protective role in HNEC infection by *S. aureus*.

### 3.6 hBD-2 abrogates *S. aureus*-induced barrier function disruption

To examine the effect of hBD-2 on the *S. aureus*-induced barrier dysfunction, the HNECs were pre-incubated with or without hBD-2 before stimulation with USA500 for 4 h. The effects of hBD-2 treatment on the barrier function of the ALI monolayer cultures of HNECs were assessed using TEER and FITC–dextran flux. When compared with the control group, infection of HNECs with USA500 induced a concentration-dependent decrease of TEER and an increase in the dextran flux, suggesting loss of epithelial integrity (**Figure 4C**). Treatment with hBD-2 of USA500-infected HNECs induced a higher TEER and a lower dextran flux compared with USA500 infection without hBD-2 pre-incubation (**Figure 4D**), indicating that hBD-2 partially abrogated the *S. aureus*-induced barrier dysfunction in HNECs. In summary, it was verified that *S. aureus* decreased the nasal epithelial barrier function, whereas hBD-2 treatment could partially reverse this effect.

### 3.7 hBD-2 attenuates *S. aureus*-induced disruption of tight junctions

**Figure 5:**
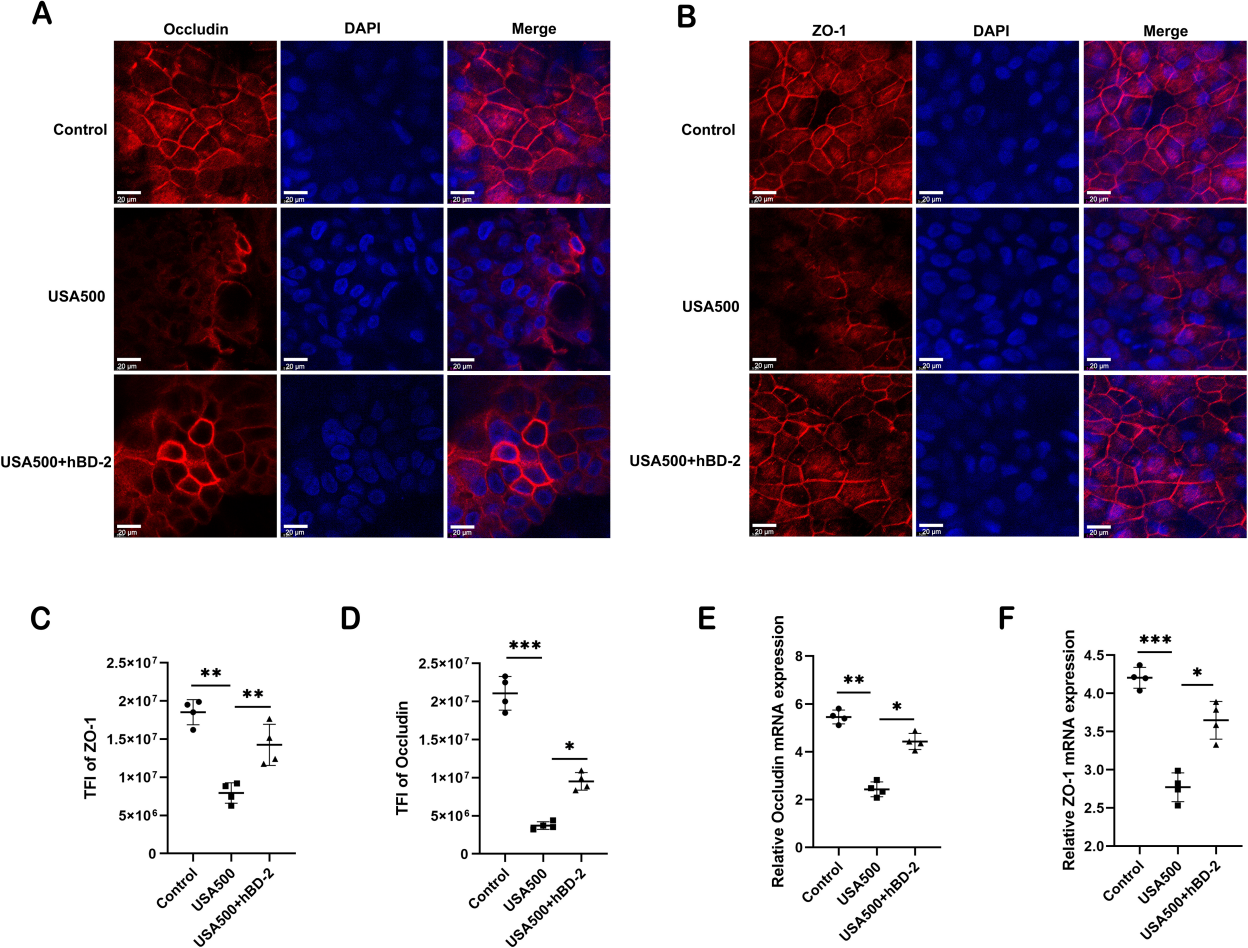
Human beta defensin-2 (hBD-2) attenuates the *Staphylococcus aureus*-induced disruption of tight junctions. **(A, B)** Representative immunofluorescence images of occludin **(A)** and ZO-1 **(B)** protein production (*red color*) in human nasal epithelial cells (HNECs) after treatment. **(C, D)** Quantified total fluorescence intensity (TFI) of occludin **(C)** and ZO-1 **(D)**. **(E, F)** Relative mRNA expression of occludin **(E)** and ZO-1 **(F)**. Data are expressed as the mean ± SD. *p < 0.05, **p < 0.01, ***p < 0.001. *TFI*, total fluorescence intensity. *Scale bar*, 5 μm.

The epithelial integrity is largely maintained by intercellular junctions called TJs. To assess whether *S. aureus* altered the epithelial integrity through the regulation of TJs, immunofluorescence staining of representative TJ proteins (occludin and ZO-1) of HNECs was conducted upon *S. aureus* USA500 infection. The immunofluorescence staining for occludin and ZO-1 showed a classic lattice-like structure between epithelial cells in the control. After USA500 infection, the structure of mucosal TJs was severely disrupted and the epithelial cells had a loose morphology, whereas hBD-2 treatment partially restored the TJ and cell morphology (**Figures 5A, B**). Quantification of TFI showed that hBD-2 significantly decreased the disruption of the junctional structure caused by USA500 (*p* < 0.05 and *p* < 0.01, respectively) (**Figures 5C, D**), indicating that hBD-2 protected the epithelial integrity by upregulating the expression of TJs. Furthermore, it was found that hBD-2 significantly increased the mRNA expression of occludin and ZO-1 in HNECs after USA500 infection (*p* < 0.05 and *p* < 0.01, respectively) (**Figures 5E, F**). In summary, hBD-2 may partially attenuate the reduction in TJs induced by *S. aureus*, suggesting the protective effect of hBD-2 on *S. aureus* infection.

## 4 Discussion

Microbes are important components in the nasal cavity, of which dysbiosis can lead to the initiation of CRS (Copeland et al., 2018; Liang et al., 2023). In particular, *S. aureus* has been widely reported to impact CRS in many aspects, including the formation of biofilms and the production of various virulence factors. Microbial virulence factors can exert their influence on the inflammatory process of CRS through several mechanisms, such as directly influencing and driving the inflammatory process (Vickery et al., 2019); facilitating the adherence and invasion into nasal epithelial cells, enabling colonization and infection (Dinges et al., 2000); and disrupting the structure and function of the mucosal barrier (Malik et al., 2015). With regard to the mucosal barrier, Murphy et al. reported a defective epithelial barrier and a decreased TJ protein expression in CRSwNP (Murphy et al., 2018). Interestingly, an association has also been demonstrated between *S. aureus* infection and decreased TJ proteins (Altunbulakli et al., 2018). In line with these studies, we found that *S. aureus* infection induced the epithelial barrier function disruption in CRSwNP, with a decrease in TEER and an increase in the dextran flux of the ALI monolayer cultures of polyp epithelial cells after *S. aureus* infection. At the same time, it was associated with changes in the TJ proteins. Specifically, the localization of ZO-1 and occludin in the cell membranes was decreased after *S. aureus* stimulation. Hence, barrier function dysfunction and loss of barrier integrity are thought to play a crucial role in the pathogenesis of CRS.

Human defensins, which are important antimicrobial and immunomodulatory peptides, exhibit broad-spectrum antimicrobial activity encompassing Gram-positive and Gram-negative bacteria, fungi, viruses, and parasites (Cieslik et al., 2021). To date, six hBDs have been identified and characterized (Schneider et al., 2005). Recent research has shown that hBDs are crucial for the mucosal immunity of the paranasal sinuses, where they initiate the immune response and engage in bactericidal action to defend against infection (Bhat et al., 2007). A multiple and wide influence of hBD-2 has been postulated in various diseases of the respiratory tract (MacRedmond et al., 2005). Recent studies have indicated significant associations in the mutations of hBD-2-encoding genes (*DEFB4A* and *DEFB4B*) with the development of atopic asthma (Borchers et al., 2021).

Several groups have observed the presence of hBDs within the secretions, tissues, and epithelial cells of patients with CRS (Hirschberg et al., 2016). However, the findings with regard to their expression levels remain inconclusive. For instance, some studies have demonstrated elevated expression of hBD-1, hBD-2, and hBD-4 in the sinonasal tissues of patients with CRSwNP compared with healthy controls (Hirschberg et al., 2016; Lee et al., 2002). hBD-2 can also induce the chemotaxis of immune cells and the activation of Toll-like receptors (TLRs) (Hirschberg et al., 2016; Lee et al., 2002). This is supported by the observation that the mRNA levels of hBD-2 are increased in the sinonasal epithelial cells from patients with CRSwNP upon stimulation with a TLR9 agonist (Ramanathan et al., 2008). In contrast, some studies reported that the expression of hBD-2 is lower in CRSwNP than in non-eosinophilic polyps associated with cystic fibrosis (Claeys et al., 2003) and that hBD-2 is upregulated in the sinus lavage fluid from patients with CRSsNP (Carothers et al., 2001).

In this study, it was demonstrated that the mRNA and protein expression levels of hBD-2 were increased in CRSwNP compared with healthy controls. Furthermore, an elevated hBD-2 protein expression positively correlated with peripheral blood neutrophils and negatively correlated with the Lund–Mackay CT score. These results suggest that the increased hBD-2 might be related to the neutrophilic inflammation in CRSwNP. Thus, hBD-2 may have a prominent role in the innate immune response of the respiratory tract. As the expression of hBDs is induced in response to the above microorganisms or their products, the upregulation of hBD in CRSwNP is not surprising.

hBD-2 is inducibly expressed across diverse epithelial cells, predominantly in response to microbial stimuli or upon exposure to pro-inflammatory cytokines, such as lipopolysaccharide, tumor necrosis factor alpha (TNF-α), and interleukin 1b (IL-1b) (Niyonsaba et al., 2006). The expression of hBD-2 is tightly regulated by pathogen-associated molecular patterns (PAMPs) through TLR signaling pathways, especially TLR2 and TLR4 (Niyonsaba et al., 2006). While hBD-2 itself is not a canonical neutrophil chemoattractant, the interaction between hBD-2 and TLR4 triggers the activation of NF-κB, driving downstream cytokine production, which can drive neutrophil chemotaxis and immune cell recruitment (Steubesand et al., 2009). These coordinated actions highlight the pivotal role of hBD-2 in bridging the innate defense mechanisms with adaptive immune activation.

Hence, the impact of hBD2 on the growth curve of *S. aureus* was also preliminarily explored. It was found that hBD-2 inhibited the growth of *S. aureus* and that the different concentrations caused growth delay. Several human defensins have been reported to induce inhibition of growth or the killing of various bacteria and fungi (Cobo and Chadee, 2013). However, to the best of our knowledge, there are no reports on the activity of hBD-2 against *S. aureus.* As an antimicrobial mechanism, defensins disrupted the membrane permeability at concentrations that correlated with the inhibition of growth. Overall, this result further demonstrates the antimicrobial effect of hBD-2 against *S. aureus* infection.

Upon *S. aureus* invasion, hBD-2 reduced its invasiveness, assessed as adhesion and internalization and its ability to cross the HNECs. The invasion assays on HNECs demonstrated that hBD-2 not only markedly inhibited the internalization of *S. aureus* but also impeded its penetration through the monolayer. These effects could have resulted from the reinforcement of the barrier integrity, as evidenced by the increased dextran flux and the decreased TEER values. Furthermore, immunofluorescence staining of TJs directly showed that treatment with hBD-2 reversed the effect of the *S. aureus*-induced epithelial barrier function disruption through the upregulation of the TJ proteins. However, this reversible effect was not sufficient to recover the mucosal epithelium. There are two possible reasons for this. The concentration of hBD-2 was determined as 50 ng/ml in the invasion assay to ensure the growth of *S. aureus*, which in turn might have decreased the protective effect of the epithelial barrier function. In summary, hBD-2 treatment of HNECs partially reversed the epithelial barrier dysfunction, suggesting the protective effect of hBD-2 on *S. aureus* infection. However, this reversible effect was not sufficient to recover the mucosal epithelium.

Notably, our group demonstrated an impaired TJ barrier and a decreased TJ protein expression in patients with CRSwNP. A compromised epithelial barrier integrity within the sinonasal mucosa is a critical factor in the pathogenesis of CRSwNP. This impaired barrier function facilitates microbial colonization and contributes to the development and persistence of chronic inflammation. Therefore, future studies should take into account the dose effect of hBD-2 and the potential contributions of other hBDs in CRS.

This study has several limitations. One of the major limitations is that, given the limited number of studies investigating the correlation between hBDs and CRS, further research is warranted to elucidate the precise role of hBDs in the pathogenesis and potential therapeutic implications of each CRS phenotype. Another limitation is that this study preliminarily demonstrated that hBD-2 abrogates the *S. aureus*-induced epithelial barrier function disruption; however, further *in vivo*/*in vitro* knockout studies were not carried out to identify the signaling pathways.

## Data Availability

The original contributions presented in the study are included in the article/**Supplementary Material**. Further inquiries can be directed to the corresponding authors.

## Supplementary material

Supplementary Figure S1 The effect of hBD-2 on the expression of the virulence-associated genes. The expression of *hla* and *coa* were significantly downregulated in the USA500 strain treated with hBD-2.

Supplementary Figure S2 The interaction between USA 500 and HNECs in the presence or absence of hBD-2. HNECs were preincubation with hBD-2 for 48 hours, then removed hBD-2. HNECs were infected with the USA 500 for 4 hours. The large circular structures indicate cell nuclei and the small punctate signals indicate USA500, indicating bacterial adhesion.

Supplementary Figure S3 HNECs collected from the inferior turbinate from healthy subjects (n=4) were cultured ex vivo at the ALI, and TEER was measured from day 3 to 27. Barrier integrity plateaued at 24 days.

Supplementary Table S1 Primer sequences used for PCR (indicated as 5′–3′).
