# Supplementary figures and images for: Human beta defensin-2 protects the epithelial barrier during methicillin-resistant Staphylococcus aureus infection in chronic rhinosinusitis with nasal polyps
Source: Front Cell Infect Microbiol. 2025 May 9;15:1551080. doi: 10.3389/fcimb.2025.1551080 (PMC12098561; doi:10.3389/fcimb.2025.1551080)

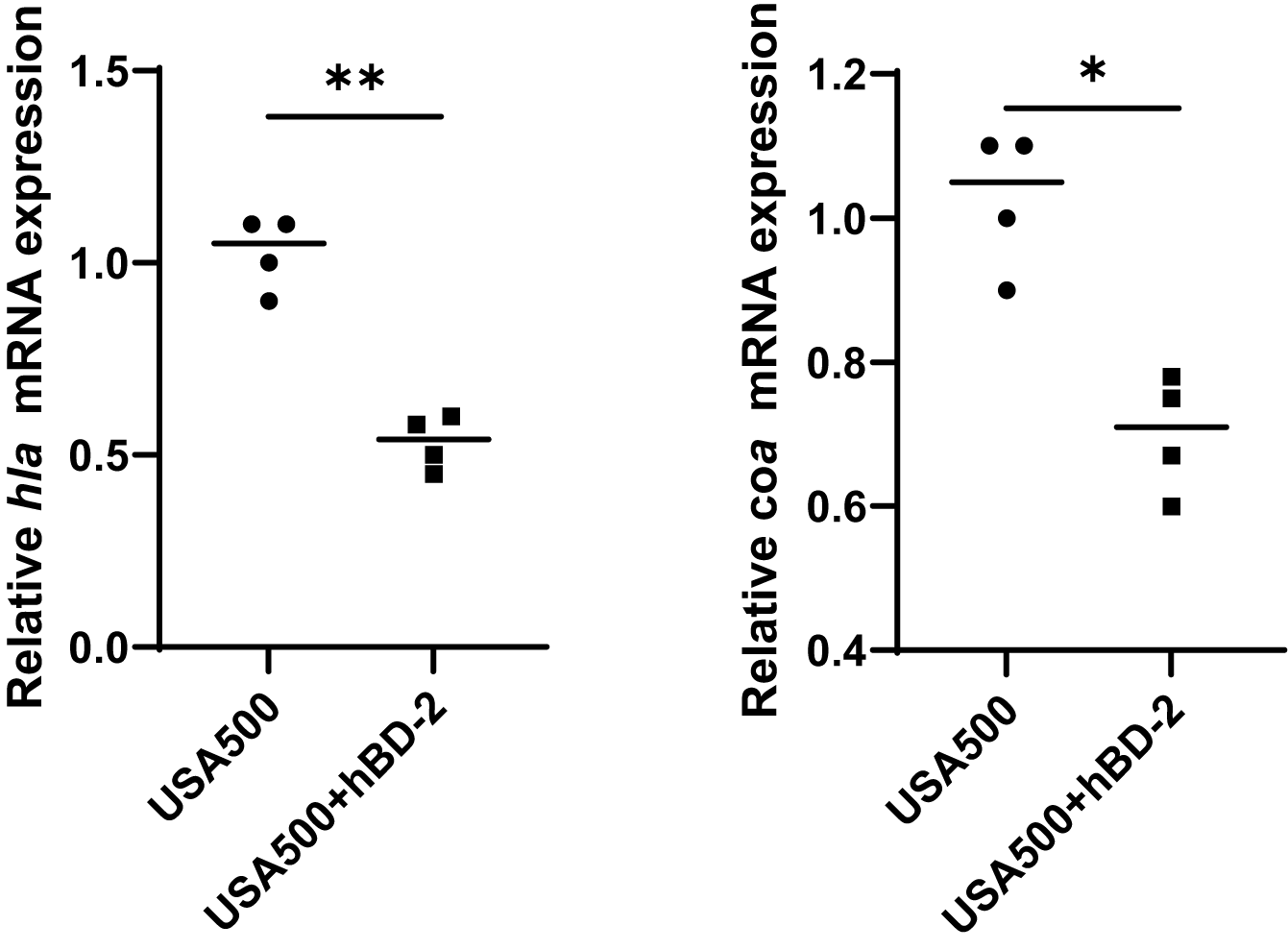

Supplement: Supplementary Figure S1 — The effect of hBD-2 on the expression of the virulence-associated genes. The expression of hla and coa were significantly downregulated in the USA500 strain treated with hBD-2. [file Image1.tif]

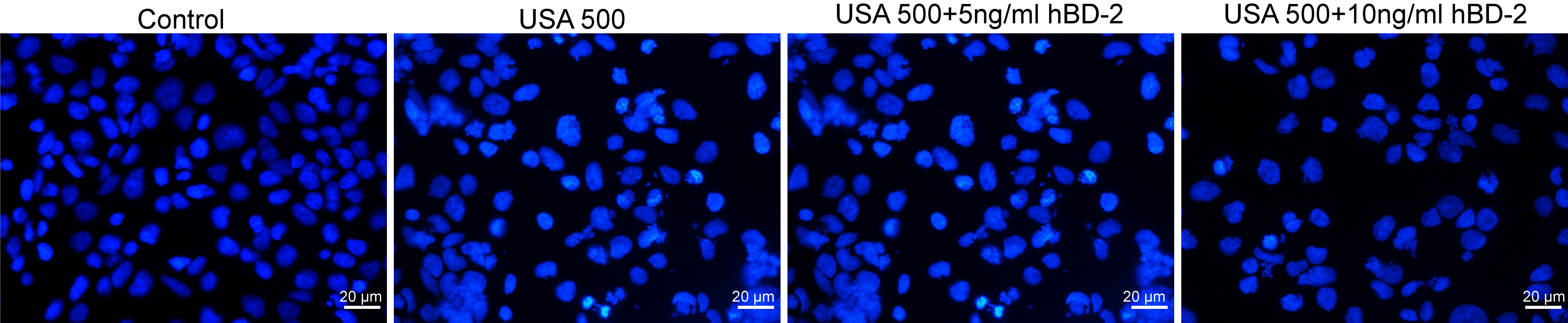

Supplement: Supplementary Figure S2 — The interaction between USA 500 and HNECs in the presence or absence of hBD-2. HNECs were preincubation with hBD-2 for 48 hours, then removed hBD-2. HNECs were infected with the USA 500 for 4 hours. The large circular structures indicate cell nuclei and the small punctate signals indicate USA500, indicating bacterial adhesion. [file Image2.tif]

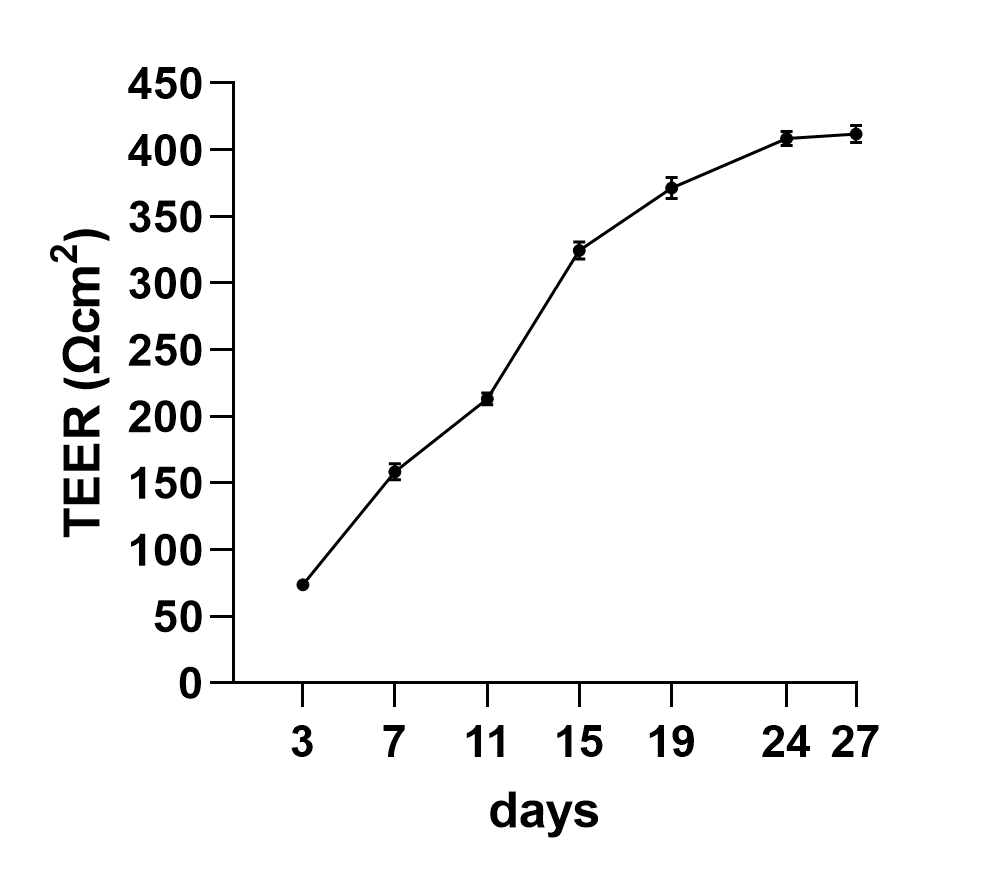

Supplement: Supplementary Figure S3 — HNECs collected from the inferior turbinate from healthy subjects (n=4) were cultured ex vivo at the ALI, and TEER was measured from day 3 to 27. Barrier integrity plateaued at 24 days. [file Image3.tif]
