# Supplementary material for: Human beta defensin-2 protects the epithelial barrier during methicillin-resistant Staphylococcus aureus infection in chronic rhinosinusitis with nasal polyps
Source: Front Cell Infect Microbiol. 2025 May 9;15:1551080. doi: 10.3389/fcimb.2025.1551080 (PMC12098561; doi:10.3389/fcimb.2025.1551080)
Supplement: Supplementary Table S1 — Primer sequences used for PCR (indicated as 5′–3′). [file Table1.docx]

TABLE S1. Primer sequences used for PCR (indicated as 5ʹ -3 ʹ)

| Gene | Forward primer | Reverse primer |
| --- | --- | --- |
| hBD-2 | CATGAGGGTCTTGTATCTCCTCT | CCTCCTCATGGCTTTTTGCAGC |
| GAPDH | ACAGTTGCCATGTAGACC | TTTTTGGTTGAGCACAGG |
| *hla* | GGTATATGGCAATCAAC | CTCGTTCGTATATTACATCT |
| *coa* | CGAGACCAAGATTCAACAAG | AAAGAAAACCACTCACATCA |
| *gyrB* | ACATTACAGCAGCGTATTAG | CTCATAGTGATAGGAGTCTTCT |
